# Serpinb1a suppresses osteoclast formation

**DOI:** 10.1016/j.bbrep.2021.101004

**Published:** 2021-04-26

**Authors:** Masayoshi Ishida, Naoyuki Kawao, Yuya Mizukami, Yoshimasa Takafuji, Hiroshi Kaji

**Affiliations:** Department of Physiology and Regenerative Medicine, Kindai University Faculty of Medicine, 377-2 Ohnohigashi, Osakasayama, Osaka, 589-8511, Japan

**Keywords:** Serpinb1, Serine protease inhibitor, Osteoclast, Osteoblast, Mechanical stress

## Abstract

Serpinb1a, a serine protease inhibitor family protein, has been implicated in immunoregulation and several metabolic disorders, such as diabetes and obesity; however, its roles in bone remain unknown. Therefore, we herein investigated the physiological functions of Serpinb1a in osteoclastic and osteoblastic differentiation using mouse cell lines. Serpinb1a overexpression markedly reduced the number of tartrate-resistant acid phosphatase (TRAP)- and calcitonin receptor-positive multinucleated cells increased by receptor activator nuclear factor κB ligand (RANKL) in mouse preosteoclastic RAW 264.7 cells. Moreover, it significantly decreased the mRNA levels of nuclear factor of activated T-cells, cytoplasmic 1 (NFATc1), TRAP and cathepsin K in these cells. Regarding osteoblasts, Serpinb1a overexpression significantly reduced the mRNA levels of alkaline phosphatase (ALP) and osteocalcin as well as ALP activity induced by bone morphogenetic protein-2 (BMP-2) in mouse mesenchymal ST2 cells, although it did not alter osteoblast differentiation in mouse osteoblastic MC3T3-E1 cells. Concerning the pathophysiological relevance of Serpinb1a, Serpinb1a mRNA levels were decreased in the soleus and gastrocnemius muscles of mice 4 weeks after bilateral sciatic nerve resection. In conclusion, we herein revealed for the first time that Serpinb1a inhibited osteoclast formation induced by RANKL in RAW 264.7 cells and suppressed BMP-2-induced ALP activity in ST2 cells.

## Highlights

• Serpinb1a overexpression markedly suppressed TRAP- and calcitonin receptor-positive osteoclast-like cell formation.
• Serpinb1a overexpression significantly suppressed the mRNA levels of bone resorption-related genes in these cells.
• Serpinb1a overexpression did not affect osteoblast differentiation in mouse osteoblastic cells.
• Serpinb1a mRNA levels were reduced in the soleus and gastrocnemius muscles of mice after bilateral sciatic nerve resection.
• Serpinb1a may be one of the crucial protease inhibitors related to unloading-induced muscle atrophy and osteoporosis.

## 1 Introduction

The serine protease inhibitor family, which includes 16 types of serpins, are important protease inhibitors [1,2]. Serpins are ubiquitously expressed in various tissues and have been implicated in pathophysiological states. Serpinb1a, a member of this family, was initially shown to play a role in innate immunity, and its involvement in the regulation of host innate immunity through the inhibition of neutrophil elastases has since been extensively examined [[3], [4], [5]]. A previous study reported that lung defenses were preserved by enhancements in neutrophil elastases in Serpinb1-deficient mice infected with *Pseudomonas aeruginosa* [6]. Moreover, proteinase 3-dependent caspase cleavage modulated neutrophil death and inflammation through the inhibition of caspase activation by Serpinb1 [7,8]. On the other hand, Takebayashi et al. showed that plasma Serpinb1 levels were correlated with insulin resistance and fat mass in patients with type 2 diabetes and obesity [9]. Although the roles of Serpinb1 in the diabetic state currently remain unclear, Serpinb1a was shown to directly promote the proliferation of pancreatic islet β cells [10,11]. These findings suggest that Serpinb1a exhibits various pathophysiological functions through the inhibition of serine protease or mechanisms other than its function as an endogenous serine protease inhibitor.

Some serpins have been shown to function in bone. Plasminogen activator inhibitor-1 (PAI-1) (SerpinE1), an inhibitor of plasminogen activators, is involved in the pathophysiology of various bone metabolic disorders through its negative effects on bone tissues as an adipokine [12]. We previously reported that PAI-1 contributed to glucose intolerance, osteopenia, delayed bone repair after bone injury and muscle wasting induced by diabetes or excess glucocorticoid levels in mice [[13], [14], [15], [16], [17]]. Moreover, a PAI-1 deficiency was shown to inhibit the differentiation of mesenchymal stem cells into osteoblasts in mice [18]. We recently revealed that Serpina3n, which is expressed in mouse osteoblasts in female-dominant manner, suppresses the osteoblast phenotypes, such as type 1 collagen expression and alkaline phosphatase (ALP) activity in mouse differentiated osteoblasts [19]. These findings suggest that serpins play significant physiological and pathophysiological roles in bone; however, the function of Serpinb1a currently remains unknown.

In the present study, we investigated the effects of Serpinb1a on mouse osteoblasts and osteoclasts using a cell line and Serpinb1a expression vector. Moreover, we tried to explore some physiological significance of Serpinb1a actions on bone related to the muscle/bone interactions.

## 2 Materials and methods

### 2.1 Cell culture

Mouse osteoblastic MC3T3-E1 cells (provided by Dr. Kodama, Ohu Dental Collage, Koriyama, Japan) were cultured in α-minimum essential medium (MEM) with 10% fetal bovine serum (FBS) and 1% penicillin/streptomycin. ST2 cells (RIKEN, Tsukuba, Japan), a mouse mesenchymal cell line, were maintained in RPMI1640 with 10% FBS and 1% penicillin/streptomycin. RAW 264.7 cells, a mouse monocyte-macrophage cell line (ATCC, Manassas, VA), were cultured in high glucose Dulbecco's Modified Eagle's Medium (DMEM) containing 10% FBS and 1% penicillin/streptomycin.

### 2.2 Quantitative reverse transcription-polymerase chain reaction (qRT-PCR)

Total RNA was extracted from cells using TriSure reagent (Nippon Genetics, Tokyo, Japan) in accordance with the manufacturer's instructions. The ReverTra Ace qPCR RT Master Mix with a gDNA Remover (Toyobo, Osaka, Japan) was used for the reverse transcription reaction. SYBR Green-based real-time PCR was performed using a StepOnePlus Real-Time PCR System (Applied Biosystems, Thermo Fisher Scientific, Waltham, MA, USA) with the THUNDERBIRD qPCR Mix (Toyobo) and specific primers. Each PCR primer set used in the present study is shown in Supplemental Table 1. mRNA expression levels were normalized to the relative amount of the housekeeping gene, glyceraldehyde-3-phosphate dehydrogenase (GAPDH).

### 2.3 DNA construction

The coding region of murine Serpinb1a was amplified by PCR with the KAPA HiFi DNA Polymerase enzyme (KAPA Biosystems, Wilmington, MA, USA) using cDNA from the mouse neonatal liver as a template and primers (5′-ATCAAGCTAGCATGGAGCAGCTGAGTTCAGC-3′ as the forward primer and 5′-ATCAACTCGAGCTATGGGGAACAAACCCTGC-3′ as the reverse primer). Serpinb1a cDNA was inserted into the mammalian expression vector, pcDNA3.1(+) (Invitrogen, Thermo Fisher Scientific) at the *Nhe*I/*Xho*I sites and the sequence was verified.

### 2.4 Gene transfection

Regarding transient transfection, the Serpinb1a expression vector was transfected into MC3T3-E1 cells, ST2 cells and RAW 264.7 cells using the jetPRIME reagent (Polyplus-transfection SA, Illkirch, France) according to the manufacturer's protocol. Transfection efficiency was confirmed with qRT-PCR for Serpinb1a for each experiment.

### 2.5 Osteoclast formation

RAW 264.7 cells transiently expressing Serpinb1a or the empty vector were seeded onto 96-well plates at 1000 cells/well. To achieve osteoclast differentiation, cells were cultured with αMEM, 10% FBS and 50 ng/ml of receptor activator nuclear factor κB ligand (RANKL) (FUJIFILM Wako Pure Chemicals, Osaka, Japan) for 6 days. Cells were then fixed with 4% formaldehyde, and tartrate-resistant acid phosphatase (TRAP) staining was performed with the TRAP Stain kit (FUJIFILM Wako Pure Chemicals) according to the manufacturer's protocol. TRAP-positive multinucleated cells (MNCs) with three and more nuclei were counted as osteoclasts. Immunocytochemistry for the calcitonin receptor was performed as previously described [20]. Briefly, cells were fixed in 10% formaldehyde at room temperature for 20 min. Cells were depleted of endogenous peroxidase activity using 3% H_2_O_2_ for 15 min, blocked with 5% FBS for 60 min, and then incubated with a mouse monoclonal anti-calcitonin receptor (CTR) antibody (Clone 2F7, Cat# sc-293299, Santa Cruz Biotechnology, Santa Cruz, CA, USA) at 4 °C overnight. Cells were incubated with a horseradish peroxidase (HRP)-conjugated anti-mouse IgG antibody (Cat#58802S, Cell Signaling Technology, Beverly, MA, USA) at room temperature for 60 min followed by 3, 3′ diaminobenzidine tetrahydrochloride (DAB) substrate (TaKaRa Bio, Otsu, Japan) according to the manufacturers' protocols. Images of samples were captured using a KEYENCE photomicroscope (original magnification, × 100). CTR-positive MNCs with three and more nuclei were counted as osteoclasts.

### 2.6 ALP activity

Samples were centrifuged at 15000 rpm at 4 °C for 10 min and ALP activity in supernatants was analyzed using the Lab assay ALP kit (FUJIFILM Wako Pure Chemicals). Total protein concentrations were measured using the Protein Assay BCA Kit (FUJIFILM Wako Pure Chemicals), as described previously [19]. Absorbance was measured at 405 nm (for ALP activity) and 562 nm (for the BCA assay) by the Multiskan Go microplate spectrophotometer (Thermo Fisher Scientific). ALP activity was defined as [units/total protein (μg)].

### 2.7 Sciatic-neurectomized (SNX) mice

Animal experiments were performed according to the guidelines of the National Institutes of Health and the institutional rules for the use and care of laboratory animals at Kindai University. All animal experiments were approved by the Experimental Animal Welfare Committee of Kindai University (Permit number: KAME-27-029). All efforts were made to minimize suffering. Mice were euthanized with excess isoflurane. SNX or sham surgery was performed on 7-week-old male C57BL/6J mice, as previously described [21].

### 2.8 Statistical analysis

Data were expressed as the mean ± the standard error of the mean (SEM). Results represent experiments performed independently three times. The significance of differences was evaluated using the Mann–Whitney *U* test for comparisons of 2 groups. Two-way analyses of variance followed by the Tukey–Kramer test were conducted for multiple comparisons. The significance level was set at P < 0.05. All statistical analyses were performed using GraphPad PRISM 7.00 software.

## 3 Results

### 3.1 Effects of Serpinb1a on osteoclast formation

**Fig. 1:**
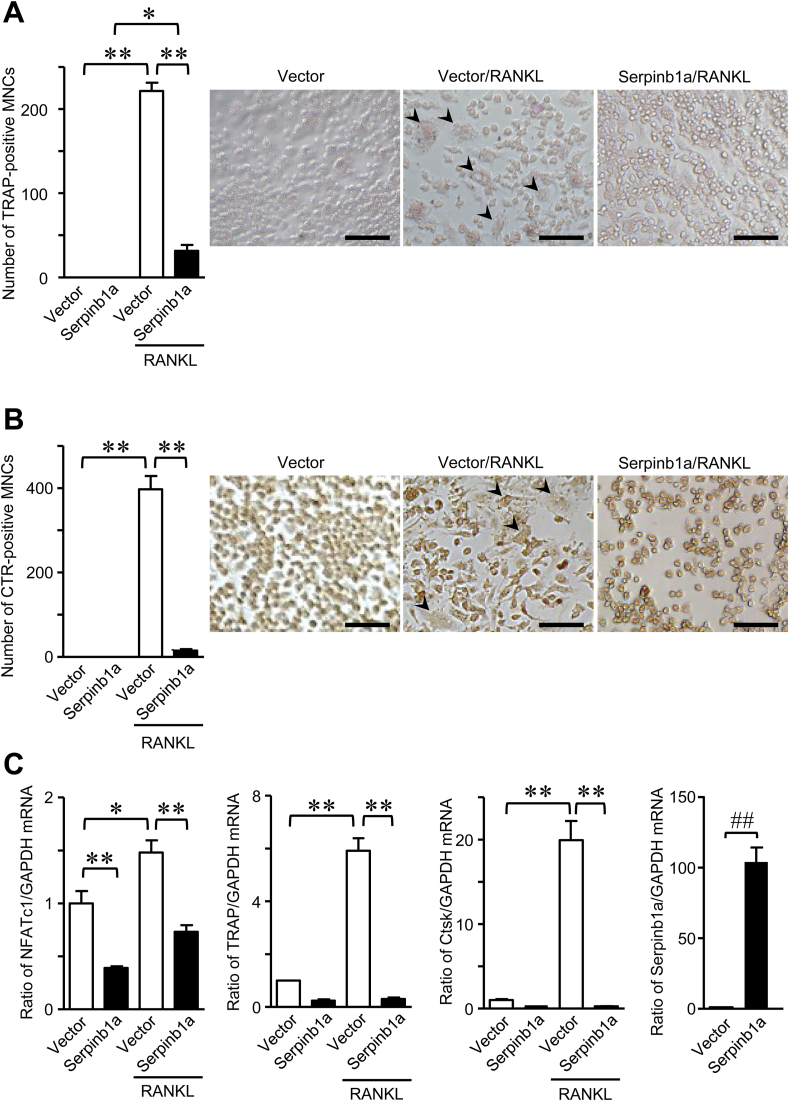
**Effects of Serpinb1a on osteoclast formation.** RAW 264.7 cells transiently expressing Serpinb1a or an empty vector were cultured with αMEM, 10% FBS and 50 ng/ml RANKL for 6 days. (A) The number of TRAP-positive MNCs was counted. (B) The number of CTR-positive MNCs was counted. Data shows the representative images (A, B). Scale bar indicates 50 μm (A, B). Arrowheads indicates TRAP- or CTR-positive MNCs (A, B). (C) Total RNA was extracted, and qRT-PCR for NFATc1, TRAP, cathepsin K (Ctsk), Serpinb1a or GAPDH was performed. Data are expressed relative to GAPDH mRNA. Data represent the mean ± SEM (n = 4 for the empty vector or Serpinb1a without RANKL group and 8 for the empty vector or Serpinb1a with RANKL group). **p < 0.01, *p < 0.05 by the Tukey-Kramer test. ^##^p < 0.01 by the Mann-Whitney *U* test.

We examined the effects of Serpinb1a on osteoclast formation using mouse preosteoclastic RAW 264.7 cells. The number of TRAP-positive MNCs enhanced by RANKL was significantly lower in RAW 264.7 cells transiently overexpressing Serpinb1a than in empty vector-transfected cells (Fig. 1A). CTR is well known as one of osteoclast-specific marker [22]. Since Serpinb1a may influence TRAP activity in MNCs, we examined CTR-positive MNCs to assess osteoclast numbers. As shown in Fig. 1B, the number of CTR-positive MNCs induced by RANKL was markedly lower in RAW 264.7 cells transiently overexpressing Serpinb1a than in empty vector-transfected cells. Moreover, the mRNA levels of NFATc1, TRAP and cathepsin K induced by RANKL were lower in RAW 264.7 cells transiently overexpressing Serpinb1a than in empty vector-transfected cells (Fig. 1C).

### 3.2 Effects of Serpinb1a on osteoblast differentiation

**Fig. 2:**
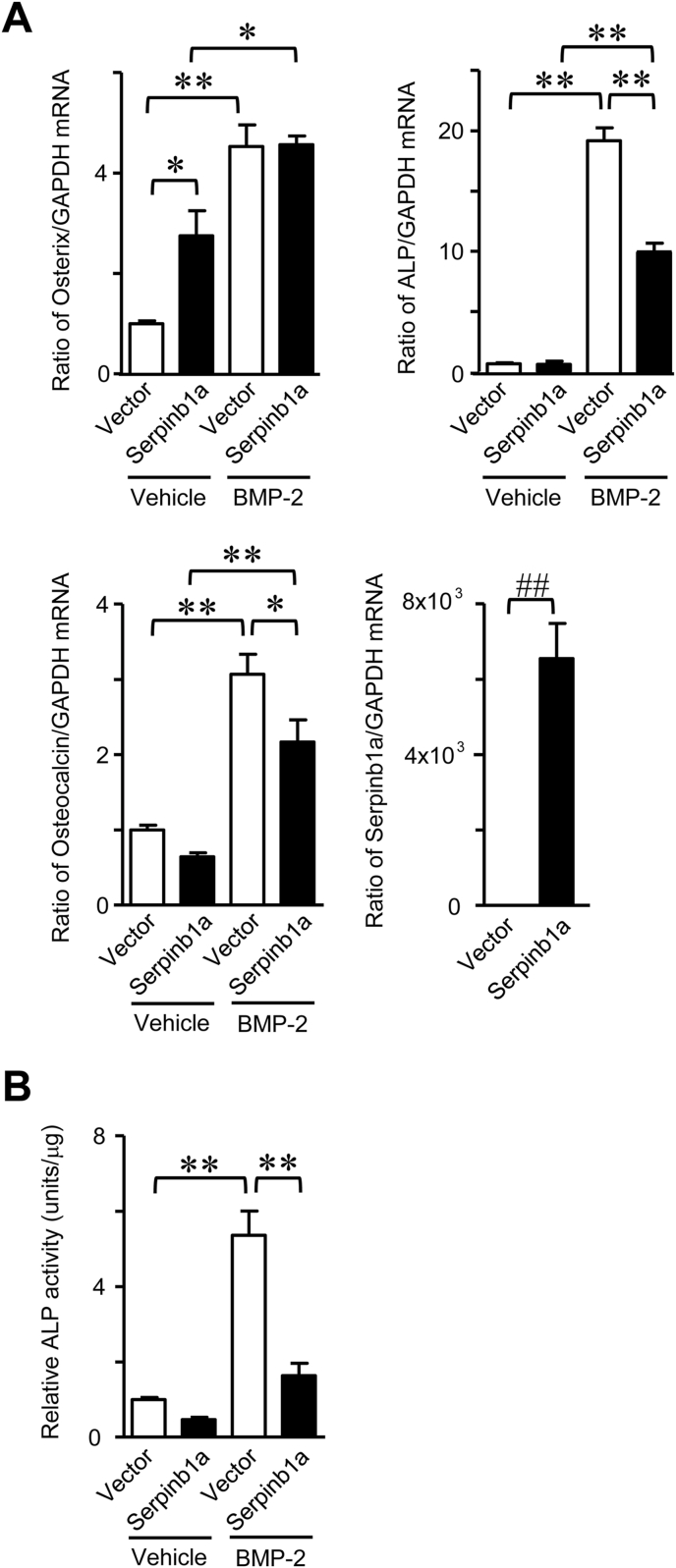
**Effects of Serpinb1a on osteoblastic differentiation.** (A) Total RNA was extracted from transiently empty vector- or Serpinb1a-transfected ST2 cells with vehicle (0.5% sucrose, 2.5% glycine, 5 mM glutamic acid, 5 mM sodium chloride and 0.01% Tween 80) or 200 ng/ml BMP-2 treatment for 72 h. Real-time PCR for Osterix, ALP, osteocalcin or GAPDH was performed. Data represent the mean ± SEM (n = 6 in each group). **p < 0.01, *p < 0.05 by the Tukey-Kramer test. ^##^p < 0.01 by the Mann-Whitney *U* test. (B) ALP activity was measured in Serpinb1a- or empty vector-transfected ST2 cells with vehicle (0.5% sucrose, 2.5% glycine, 5 mM glutamic acid, 5 mM sodium chloride and 0.01% Tween 80) or 200 ng/ml BMP-2 treatment for 72 h, as described in the Methods. Data represent the mean ± SEM (n = 6 in each group). **p < 0.01 by the Tukey-Kramer test.

We examined the effects of Serpinb1a overexpression on osteoblastic differentiation. We utilized ST2 cells, the mouse mesenchymal cell line, to examine the effects of Serpinb1a overexpression on the differentiation of mesenchymal cells into osteoblastic cells. Transient Serpinb1a overexpression significantly suppressed the mRNA levels of ALP and osteocalcin as well as ALP activity induced by BMP-2 in ST2 cells (Fig. 2A and B), but did not affect Osterix mRNA levels induced by the BMP-2 stimulation (Fig. 2A).

**Fig. 3:**
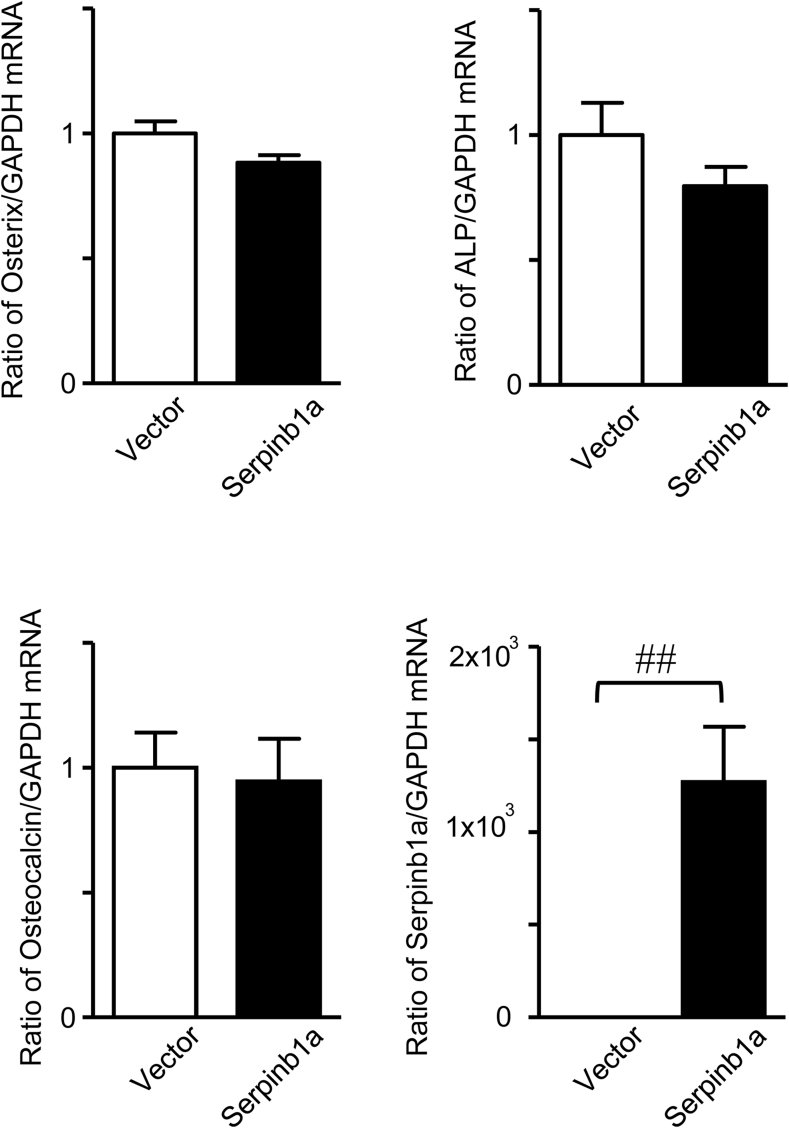
**Effects of Serpinb1a on osteoblast phenotypes.** Total RNA was extracted from transiently empty vector- or Serpinb1a-transfected MC3T3-E1 cells. Real-time PCR for Osterix, ALP, osteocalcin, Serpinb1a or GAPDH was performed. Data represent the mean ± SEM (n = 8 in each group). ^##^p < 0.01 by the Mann-Whitney *U* test.

On the other hand, transient Serpinb1a overexpression did not affect the mRNA levels of Osterix, ALP or osteocalcin in the mouse osteoblastic cell line MC3T3-E1 (Fig. 3).

### 3.3 Serpinb1a expression in skeletal muscles of SNX mice

**Fig. 4:**
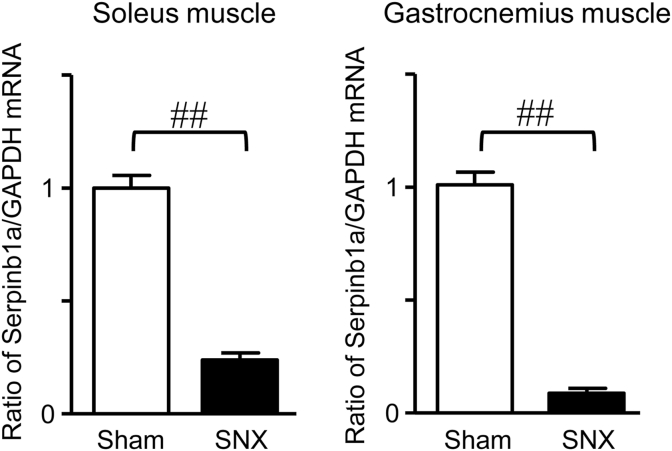
**Expression of Serpinb1a in SNX mice.** Total RNA was extracted from the soleus and gastrocnemius muscles of mice 4 weeks after SNX or sham surgery. qRT-PCR was performed for Serpinb1a or GAPDH. Data represent the mean ± SEM (n = 6 mice in each group). ^##^p < 0.01 by the Mann-Whitney *U* test.

We previously used male SNX mice to examine the effects of mechanical unloading on muscle and bone [21]. The findings obtained revealed that mechanical unloading reduced the tissue weight of the soleus muscle and trabecular bone mineral density at the tibia 4 weeks after SNX. We herein examined the expression of Serpinb1a using the soleus and gastrocnemius muscles of the same mice. As shown in Fig. 4, Serpinb1a mRNA levels in the soleus and gastrocnemius muscles were significantly lower in SNX mice than in sham-operated mice.

## 4 Discussion

Osteoclasts differentiated from monocyte-macrophage lineage cells when stimulated with RANKL, and endogenous proteases were associated with osteoclast function [23,24]. Serpinb1a has been shown to regulate the number and maturation of macrophages in mice partly through the inhibition of cathepsin and endogenous proteases [6,25,26]. In the present study, we showed that Serpinb1a overexpression decreased the numbers of TRAP- and CTR-positive MNCs as well as the expression levels of NFATc1, TRAP and cathepsin K, which are crucial osteoclast-related genes, in RAW 264.7 cells. These results indicated that Serpinb1a potently suppressed RANKL-induced osteoclast formation in RAW 264.7 cells. Therefore, we speculated that Serpinb1a may inhibit endogenous elastase in monocytes; however, the mechanisms by which Serpinb1a suppresses osteoclast formation remain unclear in our study.

In bone remodeling, osteoclastic bone resorption is followed by osteoblastic bone formation [27]. Osteoblasts are generated by the differentiation of mesenchymal stem cells or mesenchymal cells. Therefore, Serpinb1a may affect osteoblastic differentiation and osteoblast function as well as osteoclast formation. We showed that Serpinb1a overexpression significantly suppressed the expression of osteocalcin and ALP, but not Osterix, as well as ALP activity induced by BMP-2 in mouse mesenchymal ST2 cells, but did not affect the expression of Osterix, ALP or osteocalcin in mouse osteoblastic MC3T3-E1 cells. These results suggest that Serpinb1a suppressed the differentiation of mesenchymal cells into osteoblasts, but did not affect well-differentiated osteoblasts. However, we speculate that Serpinb1a may not affect the differentiation of mesenchymal cells into preosteoblasts at the early stage because Osterix is a crucial transcription factor for the commitment of preosteoblasts [28] and Serpinb1a overexpression did not affect Osterix expression induced by BMP-2 in ST2 cells.

Mullin et al. recently reported that Serpinb1 is one of 32 genes that possess a human osteoclast-specific expression quantitative trait locus, related to bone mineral density by genome-wide association studies [29]. In that study, Serpinb1 variants in osteoclasts were derived from patients with postmenopausal osteoporosis [29]. The present results suggested that Serpinb1a markedly suppressed osteoclast formation, whereas its effects on osteoblastic cells seemed to be less potent in mouse cell lines, which may lead to the increase in bone mineral density in mice. Therefore, Serpinb1a may exert positive and protective effects for bone remodeling and osteoporosis, respectively. Further *in vivo* studies are needed to clarify the roles of Serpinb1a in bone metabolism and the pathophysiology of osteoporosis.

Sarcopenia has recently been recognized as a public health issue, and the prevention and treatment of sarcopenia due to disuse or mechanical unloading in the elderly is a clinically important task. Mice with bilateral sciatic nerve resection are a useful mouse model for examining mechanical unloading-induced muscle wasting and osteopenia, as previously reported [21]. Regarding serpins, we previously showed that the expression levels of Serpina3n were higher in female than in male mouse osteoblasts, which exerted negative effects on osteoblastic function in mouse cells [19]. Moreover, Serpina3n, identified by mass spectroscopic analyses using excess glucocorticoid-treated mouse muscles, appeared to protect against muscle atrophy in Serpina3n transgenic mice [30,31]. Although the significance of Serpinb1a in the pathological states of musculoskeletal disorders and muscle/bone interactions remain unclear, these findings raised us the possibility that Serpinb1a may be related to unloading-induced muscle wasting and osteopenia. Therefore, we herein examined the expression of Serpinb1a in the soleus and gastrocnemius muscles of SNX mice, a mechanical unloading mouse model. The soleus and gastrocnemius muscles are representative muscles with type I and type IIb myofiber-dominant muscles, respectively. In the present study, mechanical unloading by SNX reduced the expression levels of Serpinb1a in both the soleus and gastrocnemius muscles of mice. This result suggested that mechanical unloading suppressed Serpinb1a expression in skeletal muscles. Taken together with the Serpinb1a-induced suppression of osteoclast formation, mechanical unloading-suppressed Serpinb1a secretion from muscles may enhance bone resorption and subsequent increases in bone mineral density in mice. Interestingly, in proteomics analyses of dystrophic muscles conducted by Arecco et al. Serpinb1a accumulated in these muscles over several weeks, and elastase levels and activity were enhanced in the dystrophic muscles of older mice [32]. Moreover, the addition of recombinant elastase markedly repressed myoblast proliferation, survival, myotube formation as well as MyoD expression. These findings suggest that Serpinb1a inhibits endogenous elastase to disturb muscle function. Xu et al. also identified Serpinb1a as one of the genes expressed in injured muscles and during muscle regeneration by cumulative cDNA microarray analyses [33].

The vitamin D receptor (VDR), a member of the nuclear receptor superfamily, plays a crucial role in the physiological effects of vitamin D [34]. The expression of many of the genes involved in not only calcium/phosphate homeostasis, but also cellular proliferation and differentiation as well as immune responses is controlled by VDR responses [34,35]. He et al. revealed that the up-regulation of Serpinb1a expression by active vitamin D inhibited elastase activity to protect against chlamydial infection in mice [36]. Furthermore, the expression of Serpinb1a was shown to be induced by active vitamin D in both human transformed and normal mammary cells using cumulative gene expression analyses with RNA-seq [37]. Moreover, Serpinb1 was identified as a vitamin D-responsive gene in both human breast malignant and normal tissues [38]. Hui et al. showed that active vitamin D inhibited human keratinocyte HaCaT cell proliferation through Serpinb1 [39]. However, we previously observed that a vitamin D deficiency did not affect Serpinb1 mRNA levels in the tibia and gastrocnemius muscles of VD-deficient diet-fed mice (data not shown) [40,41]. Therefore, VDR signaling may not be important for the roles of Serpinb1a expression in muscle/bone interactions in mice, although further studies using active vitamin D-treated mice are needed to elucidate the relationships between vitamin D and Serpinb1a in muscle and bone.

In conclusion, we herein demonstrated for the first time that Serpinb1a suppressed osteoclast formation enhanced by RANKL in RAW 264.7 cells. Moreover, it decreased BMP-2-induced ALP activity in ST2 cells. Therefore, Serpinb1a would be one of the crucial protease inhibitor in mechanical unloading-induced muscle atrophy and osteoporosis. However, the mechanisms by which Serpinb1a suppresses osteoclast formation and osteoblast differentiation have remained unclear in the present study. Further studies are necessary to clarify this issue.

## Funding

This study was partly supported by a grant from The 10.13039/100008731Nakatomi Foundation, Japan, to M.I. Grants-in-Aid for Scientific Research (C:20K09514) to H.K. and (C: 19K09659) to M.I. from the 10.13039/501100001700Ministry of Education, Culture, Sports, Science, and Technology of Japan and a Grant-in-Aid for Scientific Research on Innovative Areas (grant number 15H05935, “Living in Space”) to H.K. from the 10.13039/501100001700Ministry of Education, Culture, Sports, Science, and Technology of Japan.

## Author contributions

M.I. and H.K. contributed to the conception and design of the research. M.I., N.K., Y.M. and Y.T. performed experiments. M.I. analyzed data. M.I. and H.K. interpreted the results of the experiments. M.I. prepared figures. M.I. drafted the manuscript. M.I. and H.K. edited and revised the manuscript. All authors approved the final version of the manuscript.

## Declaration of competing interest

The authors declare that they have no known competing financial interests or personal relationships that could have appeared to influence the work reported in this paper.
