# Supplementary material for: Serpinb1a suppresses osteoclast formation
Source: Biochem Biophys Rep. 2021 Apr 26;26:101004. doi: 10.1016/j.bbrep.2021.101004 (PMC8100536; doi:10.1016/j.bbrep.2021.101004)
Supplement: Multimedia component 1 [file mmc1.doc]

**Table S1. Primers used for quantitative RT-PCR experiments.**

| Gene |  | Sequence |
| --- | --- | --- |
| Serpinb1a | Forward | TCACACTCTGAAACTTGCCAACA |
|  | Reverse | CCAAGTCAGCACCATACATCTTCT |
| TRAP | Forward | CAGCTGTCCTGGCTCAAAA |
|  | Reverse | ACATAGCCCACACCGTTCTC |
| Ctsk | Forward | GAGGGCCAACTCAAGAAGAA |
|  | Reverse | GCCGTGGCGTTATACATACA |
| NFATc1 | Forward | CAAGTCTCACCACAGGGCTCACTA |
|  | Reverse | GCGTGAGAGGTTCATTCTCCAAGT |
| Osterix | Forward | AGCGACCACTTGAGCAAACAT |
|  | Reverse | GCGGCTGATTGGCTTCTTCT |
| ALP | Forward | ATCTTTGGTCTGGCTCCCATG |
|  | Reverse | TTTCCCGTTCACCGTCCAC |
| Osteocalcin | Forward | CCTGAGTCTGACAAAGCCTTCA |
|  | Reverse | GCCGGAGTCTGTTCACTACCTT |
| GAPDH | Forward | ACGGCAAATTCAACGGCAC |
|  | Reverse | CTCCACGACATACTCAGCAC |

Serpinb1a, serine (or cysteine) peptidase inhibitor, clade B, member 1a; TRAP, tartrate-resistant acid phosphatase; Ctsk, cathepsin K; NFATc1, nuclear factor of activated T cells, cytoplasmic 1; ALP, alkaline phosphatase; GAPDH, glyceraldehyde-3-phosphate dehydrogenase.
